# Transient receptor potential genes, smoking, occupational exposures and cough in adults

**DOI:** 10.1186/1465-9921-13-26

**Published:** 2012-03-23

**Authors:** Lidwien AM Smit, Manolis Kogevinas, Josep M Antó, Emmanuelle Bouzigon, Juan Ramón González, Nicole Le Moual, Hans Kromhout, Anne-Elie Carsin, Isabelle Pin, Deborah Jarvis, Roel Vermeulen, Christer Janson, Joachim Heinrich, Ivo Gut, Mark Lathrop, Miguel A Valverde, Florence Demenais, Francine Kauffmann

**Affiliations:** 1 INSERM, CESP Centre for research in Epidemiology and Population Health, U1018, Respiratory and environmental epidemiology Team, Villejuif F-94807, France; 2 Université Paris Sud 11, UMRS 1018, Villejuif F-94807, France; 3 Division of Environmental Epidemiology, Institute for Risk Assessment Sciences, PO Box 80178, 3508 TD Utrecht, The Netherlands; 4 Centre for Research in Environmental Epidemiology (CREAL), Barcelona, Spain; 5 Municipal Institute of Medical Research (IMIM-Hospital del Mar), Barcelona, Spain; 6 CIBER Epidemiologia y Salud Pública (CIBERESP), Barcelona, Spain; 7 National School of Public Health, Athens, Greece; 8 Department of Experimental and Health Sciences, Universitat Pompeu Fabra Barcelona, Spain; 9 INSERM, U946, F-75010, Paris, France; 10 Université Paris Diderot, Sorbonne Paris Cité, Institut Universitaire d'Hématologie, Paris F-75010, France; 11 Fondation Jean Dausset-Centre d'Etude du Polymorphisme Humain (CEPH), Paris F-75010, France; 12 INSERM, U823, Grenoble, France; 13 Université Joseph Fourier-Grenoble 1, Grenoble, France; 14 Centre Hospitalier Universitaire de Grenoble, Grenoble, France; 15 Respiratory Epidemiology and Public Health Group, National Heart and Lung Institute, Imperial College, London, UK; 16 Department of Medical Sciences: Respiratory Medicine and Allergology, Uppsala University, Uppsala, Sweden; 17 Institute of Epidemiology, Helmholtz Centre, Munich, Germany; 18 Commissariat à l'Energie Atomique, Institut de Génomique, Centre National de Génotypage (CNG), Evry, France; 19 Currently Centro Nacional de Analisis Genomico, Barcelona, Spain; 20 Laboratory of Molecular Physiology and Channelopathies, Universitat Pompeu, Fabra, Barcelona, Spain

**Keywords:** Asthma, Gene-environment interaction, Irritant exposure, Smoking, TRP channel

## Abstract

**Background:**

Transient receptor potential (TRP) vanilloid and ankyrin cation channels are activated by various noxious chemicals and may play an important role in the pathogenesis of cough. The aim was to study the influence of single nucleotide polymorphisms (SNPs) in *TRP *genes and irritant exposures on cough.

**Methods:**

Nocturnal, usual, and chronic cough, smoking, and job history were obtained by questionnaire in 844 asthmatic and 2046 non-asthmatic adults from the Epidemiological study on the Genetics and Environment of Asthma (EGEA) and the European Community Respiratory Health Survey (ECRHS). Occupational exposures to vapors, gases, dusts, and/or fumes were assessed by a job-exposure matrix. Fifty-eight tagging SNPs in *TRPV1*, *TRPV4*, and *TRPA1 *were tested under an additive model.

**Results:**

Statistically significant associations of 6 *TRPV1 *SNPs with cough symptoms were found in non-asthmatics after correction for multiple comparisons. Results were consistent across the eight countries examined. Haplotype-based association analysis confirmed the single SNP analyses for nocturnal cough (7-SNP haplotype: p-global = 4.8 × 10^-6^) and usual cough (9-SNP haplotype: p-global = 4.5 × 10^-6^). Cough symptoms were associated with exposure to irritants such as cigarette smoke and occupational exposures (p < 0.05). Four polymorphisms in *TRPV1 *further increased the risk of cough symptoms from irritant exposures in asthmatics and non-asthmatics (interaction p < 0.05).

**Conclusions:**

*TRPV1 *SNPs were associated with cough among subjects without asthma from two independent studies in eight European countries. *TRPV1 *SNPs may enhance susceptibility to cough in current smokers and in subjects with a history of workplace exposures.

## Background

TRPV1, TRPV4, and TRPA1 cation channels are members of the vanilloid (TRPV) and ankyrin (TRPA) subfamily of transient receptor potential channels. These channels are expressed in different cells of the lung, including sensory neurons participating in airway reflex responses, bronchial smooth muscle, and epithelial and endothelial cells [1,2]. TRPV1 channels are activated by capsaicin, heat, particulate matter, and various noxious chemicals, and are upregulated in airway nerves and airways smooth muscle of individuals with cough [1-4]. It has recently been shown that the *TRPV1 *Ile585Val single nucleotide polymorphism (SNP) results in a loss-of-channel function, and that this SNP is associated with a lower risk of cough and wheezing among children with asthma [5]. TRPA1 acts as a receptor for a wide range of irritants and chemicals, including air pollutants and some of the principal components of cigarette smoke [6,7]. Agonists of TRPV1 and TRPA1 channels can elicit a reproducible cough response in humans [8,9], and induce neurogenic inflammatory responses in experimental models [7,10,11]. TRPV1 and TRPA1 have been put forward as major targets for novel anti-tussive drugs [12,13].

TRPV4 could play a role in the pathogenesis of airway disease through regulation of endothelial and epithelial permeability in the lungs, bronchial smooth muscle cell contractility, and mucociliary transport [14-17]. TRPV4 dysfunction has already been associated to airway diseases: airway epithelial cells from cystic fibrosis patients show a defective regulation of TRPV4 [18,19], and SNPs in the *TRPV4 *gene have been found to be associated with chronic obstructive pulmonary disease (COPD) [20].

Because of the central role of TRP channels in cough response, we hypothesized that variants in genes encoding TRP channels may be associated with cough symptoms. Moreover, given the critical involvement of TRP channels in irritant sensing, *TRP *variants may be particularly relevant for irritant-induced cough [21]. Exposures to irritants, for instance in cigarette smoke, or in exposures at work can lead to a spectrum of asthma-related symptoms, including cough [21]. In recent years, studies on asthma and chronic cough symptoms in workers exposed to irritant cleaning products have raised interest in chronic or repeated exposures to relatively low levels of irritants [22-24]. However, little is known about the mechanisms implicated in irritant-induced airway disease.

We aimed to study association of SNPs in candidate genes *TRPV1*, *TRPV4*, and *TRPA1 *with cough symptoms. In addition, we explored whether *TRP *variants modulate associations between occupational exposures and cough symptoms, or between smoking and cough symptoms. The study was carried out in adults from the French Epidemiological study on the Genetics and Environment of Asthma (EGEA) and from the European Community Respiratory Health Survey (ECRHS).

## Methods

### Study population and design

EGEA is a case-control and a family study of asthma [25]. The population comprises individuals with asthma aged 7-70 who were recruited from six chest clinics in five French cities, population-based control subjects, and relatives of asthmatic probands (either the proband's parents and siblings, or the proband's spouse and children). In the present analysis, only genetically unrelated adults aged 27-70 from the parental generation (adults with asthma and spouse, or the parents of an individual with asthma) were included, as described in detail elsewhere [26].

The methodology of ECRHS has been described elsewhere [27]. Briefly, the ECRHS is a random population-based multicentre cohort from sixteen centers (eight countries with DNA samples) of subjects aged 20-44 at time of recruitment (1990, ECRHS-I) and then followed-up approximately 10 years later (ECRHS-II). In addition to the random sample, a complementary, enriched sample of subjects with asthma symptoms at recruitment but who had not been selected at random to take part in ECRHS-II was also included in the study and follow-up. All asthma cases and a random sample of subjects without asthma were genotyped [28].

In total, 689 subjects from EGEA and 2201 subjects from ECRHS with complete questionnaire and *TRP *genotyping data were included in the present analysis (844 subjects with asthma, 2046 subjects without asthma). Additional file 1: Figures S1 and S2 present a flow chart of the study population. Ethical approval was obtained for EGEA and for each ECRHS centre from the appropriate institutional ethics committee and written consent was obtained from each participant.

### Questionnaire

Identical questions were used to assess cough symptoms, job history, and smoking in both studies [29]. Nocturnal cough was defined as a positive answer to the question "Have you been woken by an attack of coughing at any time in the last 12 months?". Usual cough was defined as a positive answer to the question "Do you usually cough first thing in the morning in the winter?" *or *"Do you usually cough during the day, or at night, in the winter?". Chronic cough was defined as usual cough *and *a positive answer to "Do you cough like this on most days for as much as three months each year?". The role of recent respiratory infections (like a common cold) in cough symptoms was studied by the question "Have you had a respiratory infection in the last 3 weeks?". The definition of (ever) asthma in both studies is given in the Additional File. Smoking was defined as described earlier [29,30], and subjects were categorized into three classes: current smokers, ex smokers and never smokers. Subjects reported their two most recent jobs, which were coded according to international standard classification of occupations (ISCO-88). Jobs were linked to low or high exposure to biological dust, mineral dust, and vapors, gases, or fumes using the ALOHA job-exposure matrix (JEM) [31]. Occupational exposure to vapors, gases, dusts, and/or fumes (VGDF) was defined as low or high exposure to any VGDF in at least one job. More details are given in the Additional File.

### Genotyping

A preliminary candidate gene study on *TRPV1 *and *TRPV4 *was conducted earlier in EGEA [32]. To enable replication, we used genome-wide genotyping of all EGEA and ECRHS subjects from the Gabriel consortium genome-wide association study [28], and we included *TRPA1 *as an additional candidate gene. All SNPs within the gene region (at least 10 kb upstream of the 5' end through 5 kb downstream of the 3' end according to the HapMap database) were selected from the Illumina Human610 quad array panel [28]. Nineteen SNPs in *TRPV1 *(located at chromosome 17p13.3), 10 SNPs in *TRPV4 *(12q24.1), and 29 SNPs in *TRPA1 *(8q13) were studied. These SNPs fulfilled the quality control criteria that have been applied to the Gabriel study (*see *Additional File) [28], and allowed us to capture the majority of common haplotype variations of the three genes (i.e., haplotype with a frequency ≥ 5%). Additional Figures E3, E4, and E5 show linkage disequilibrium (LD) between SNPs for the three genes.

### Statistical analysis

Statistical analysis is described in detail in the Additional File. The EGEA and ECRHS study populations were pooled to increase statistical power, and consistency of results was verified by analyzing both studies separately and by performing a random effects meta-analysis of EGEA and the eight ECRHS country-specific samples. The latter allowed to test for heterogeneity across samples. Analyses were conducted in subjects with and without asthma separately, because cough is associated with asthma, and EGEA subjects were recruited through asthmatic patients. The effect of single SNPs on cough was tested under an additive genetic model with the minor allele as risk allele, as was done in previous studies that have shown associations between *TRPV *SNPs and respiratory outcomes [5,20]. Subjects without the given cough symptom were studied as the reference group. Population admixture was taken into account by including informative principal components for within-Europe diversity for EGEA and ECRHS as covariates in the association analysis [28]. Age, smoking, sex, occupational exposure, study and sample (dummy indicators for EGEA, random sample ECRHS, and symptomatic sample of ECRHS) were also incorporated in the logistic model. P-values of association were corrected to address multiple testing (n = 174 tests, i.e. 58 SNPs × 3 cough outcomes) using the Benjamini-Hochberg method [33]. Haplotype association analyses were assessed using SNPassoc [34] and haplo.stats [35] R packages, and we further used an unbiased sliding window approach to construct successive and adjacent 2- to 9-SNPs haplotypes. To explore whether the effect of occupational exposure or current smoking on cough was modified by *TRP *genotype we introduced a multiplicative gene-environment interaction term in the model (SNP × occupational exposure or SNP × current smoking). The statistical significance of the interaction term was assessed by using a generalized score test which follows a chi-square distribution with 1 degree of freedom.

## Results

Personal characteristics and the prevalence of cough symptoms of the study population are shown in Table 1. Any type of cough was reported by 60.0% of subjects with asthma, and 33.7% of subjects without asthma. Among subjects with asthma, 25.8% reported both nocturnal cough and usual cough, 22.0% reported nocturnal cough but no usual cough, and 12.2% reported usual cough but no nocturnal cough. For subjects without asthma, these prevalence rates were 8.3%, 18.0%, and 7.4%, respectively. Women were less often exposed to VGDF than men, and were more often never smokers (p < 0.01). Exposure to VGDF was not associated with asthma (OR 0.99 [0.81-1.21]), and this association did not differ between men and women.

**Table 1:** Characteristics of the study population

		Asthma			No asthma	
	
	Men	Women	*P *value	Men	Women	*P *value
n Study, n (%)	368	476		1008	1038	
EGEA	107 (29.1)	96 (20.2)		248 (24.6)	238 (22.9)	
ECRHS	261 (70.9)	380 (79.8)		760 (75.4)	800 (77.1)	
Age, mean (sd) Smoking habits, n (%)	44.4 (8.3)	42.2 (7.4)	0.006	44.1 (7.7)	43.9 (7.5)	0.65
Never smokers	141 (38.3)	232 (48.7)	0.004	372 (36.9)	515 (49.6)	< 0.001
Ex smokers	130 (35.3)	125 (26.3)		278 (27.6)	237 (22.8)	
Current smokers	97 (26.4)	119 (25.0)		358 (35.5)	286 (27.6)	
≤ 16 pack-years	36 (9.8)	66 (13.9)		121 (12.0)	150 (14.5)	
> 16 pack-years	49 (13.3)	40 (8.4)		188 (18.7)	88 (8.5)	
pack-years unknown	12 (3.3)	13 (2.7)		49 (4.9)	48 (4.6)	
Exposure to VGDF, n(%)* Cough symptoms, n(%)	182 (51.0)	182 (39.8)	0.002	544 (55.0)	361 (36.0)	< 0.001
Nocturnal cough	131 (35.7)	272 (57.1)	< 0.001	226 (22.4)	314 (30.3)	< 0.001
Usual Cough	123 (33.6)	195 (41.4)	< 0.001	167 (16.6)	152 (14.7)	0.23
Chronic cough	64 (17.7)	103 (22.1)	0.12	73 (7.3)	52 (5.1)	0.03
Respiratory infection last 3 weeks, n (%)	52 (14.4)	54 (11.5)	0.21	87 (8.7)	80 (7.8)	0.57

*VGDF: vapours, gases, dust, or fumes

Overall, out of 1157 subjects with any type of cough, 550 had nocturnal only (47.5%), 150 usual cough only (13.0%), 92 chronic cough (7.9%), 165 usual and nocturnal (14.3%) and 200 chronic and nocturnal (17.3%)

Twenty-eight subjects had missing values for one of the cough variables. Forty-two subjects had missing values for the infection variable

### Occupational exposures, smoking and cough symptoms

In subjects with asthma, female gender was significantly associated with all cough symptoms, and current smoking was associated with usual cough and chronic cough (Table 2). When current smokers were stratified by median cigarette consumption (16 pack-years), the association was only observed for heavy smokers. Occupational exposure to VGDF was significantly associated with nocturnal cough (OR 1.39 [1.04-1.87]) and chronic cough (OR 1.69 [1.18-2.43]). Respiratory infection during the last 3 weeks was only significantly associated with an increased prevalence of nocturnal cough. In subjects without asthma, nocturnal cough was more prevalent among female subjects, and current smoking was associated with all cough symptoms. The association was stronger for heavy smokers but statistically significant also for light smokers. Respiratory infection was positively and significantly associated with nocturnal cough and usual cough. No association between occupational exposure and cough symptoms was found (Table 2). Although the pattern of determinants was similar in both studies, associations with occupation in asthmatics tended to be stronger in EGEA (chronic cough: OR 2.82 [1.39-5.72]) than in ECRHS (OR 1.34 [0.87-2.07])

**Table 2:** Determinants of nocturnal cough, usual cough, and chronic cough in 844 adults with asthma and in 2046 adults without asthma

	**Subjects with asthma**			**Subjects without asthma**		
	
	**Nocturnal cough**	**Usual cough**	**Chronic cough**	**Nocturnal cough**	**Usual cough**	**Chronic cough**
	
Female sex	**2.85 (2.10-3.86)**	**1.42 (1.05-1.93)**	**1.49 (1.02-2.17)**	**1.67 (1.35-2.07)**	0.95 (0.73-1.23)	0.78 (0.53-1.16)
Exposure to VGDF*	**1.39 (1.04-1.87)**	1.29 (0.96-1.73)	**1.69 (1.18-2.43)**	1.09 (0.88-1.35)	0.98 (0.75-1.27)	1.05 (0.70-1.56)
Age, per 10 y	1.17 (0.96-1.42)	1.10 (0.90-1.34)	1.16 (0.90-1.48)	1.04 (0.91-1.21)	1.10 (0.91-1.32)	1.16 (0.88-1.54)
Ex smoker vs. never smoker	0.89 (0.62-1.26)	0.86 (0.60-1.24)	0.65 (0.40-1.05)	1.11 (0.85-1.46)	0.94 (0.64-1.37)	1.07 (0.57-2.01)
Current smoker vs. never smoker	1.15 (0.80-1.66)	**2.04 (1.43-2.91)**	**2.12 (1.40-3.21)**	**1.68 (1.32-2.14)**	**3.37 (2.53-4.49)**	**4.26 (2.67-6.79)**
≤ 16 pack-years vs. never smoker†	1.03 (0.64-1.64)	1.08 (0.67-1.74)	1.13 (0.63-2.02)	1.28 (0.93-1.76)	**2.18 (1.49-3.19)**	**2.31 (1.24-4.33)**
> 16 pack-years vs. never smoker†	1.44 (0.87-2.39)	**4.45 (2.66-7.45)**	**3.71 (2.20-6.27)**	**2.34 (1.71-3.20)**	**5.18 (3.66-7.34)**	**6.43 (3.79-10.92)**
Respiratory infection last 3 weeks	**1.84 (1.18-2.87)**	1.17 (0.76-1.82)	1.13 (0.68-1.90)	**2.45 (1.74-3.44)**	**1.51 (1.00-2.29)**	1.54 (0.84-2.83)
						

Multivariate GEE model, adjusted for all variables in the table, principal components for within-Europe diversity, sample, and study; results are presented as OR (95% CI). Bold type indicates *P *< 0.05

*VGDF: vapours, gases, dust, or fumes

† in models without current smoker vs. never smoker. Coefficients for all other variables were basically similar (e.g.OR were for nocturnal cough 2.57, 1.27, 1.15, 0.89 and 1.67, 1.06, 1.01 and 1.13 for sex, VGDF, age and ex-smoker in subjects with and without asthma respectively)

### Association analysis *TRP *polymorphisms and cough

**Figure 1:**
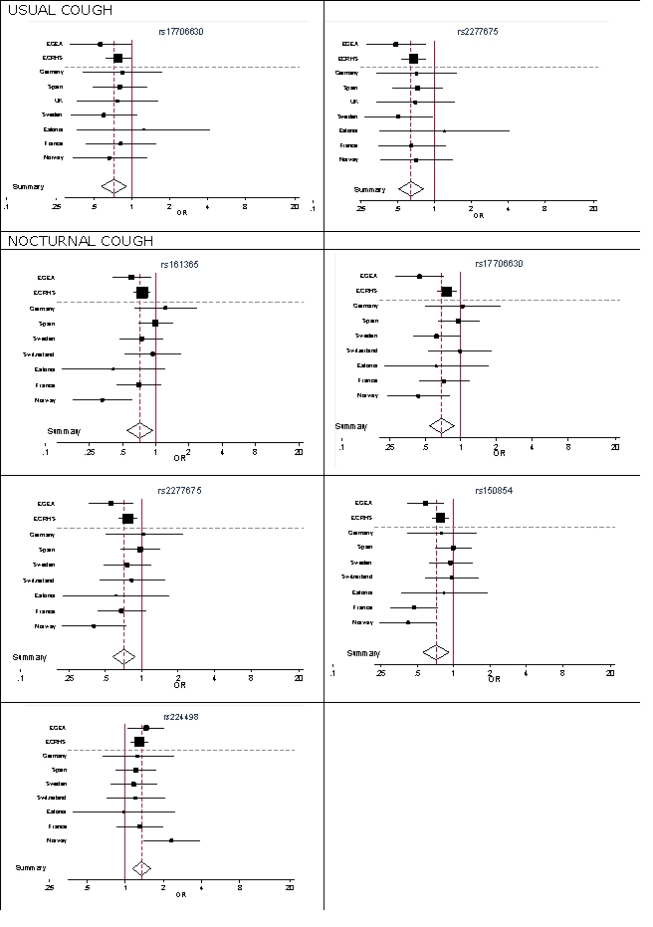
**Forest plots of odds ratios (OR) for SNPs associated with nocturnal or usual cough in both EGEA and ECRHS, according to study and country (ECRHS)**. Summary ORs were calculated using a random effects meta-analysis applied to EGEA and the separate ECRHS countries. Summary ORs (95% CI) for the association between rs17706630 and rs2277675 and usual cough were 0.72 (0.57-0.91) and 0.64 (0.51-0.81), respectively. Summary ORs (95% CI) for the association between rs161365, rs17706630, rs2277675, rs150854, and rs224498 and nocturnal cough were 0.72 (0.55-0.94), 0.69 (0.54-0.88), 0.71 (0.58-0.88), 0.72 (0.56-0.92), and 1.34 (1.14-1.58), respectively. ORs for associations with usual cough could not be estimated for Switzerland, and ORs for nocturnal cough could not be estimated for the United Kingdom (UK)

In subjects without asthma, 10 *TRPV1 *SNPs were associated with nocturnal cough (4.3 × 10^5 ^≤ p ≤ 0.044), 6 *TRPV1 *SNPs were associated with usual cough (2.0 × 10^-5 ^≤ p ≤ 0.046), and 4 *TRPV1 *SNPs were associated with chronic cough (0.005 ≤ p ≤ 0.045) (Table 3). After adjustment for multiple testing, 7 associations between *TRPV1 *SNPs and cough symptoms remained statistically significant (Table 3). All associations between *TRPV1 *SNPs and cough that were significant in the pooled analysis (after correction for multiple testing) showed the same direction of association in ECRHS and EGEA (Additional file 1: Table S1 and S2). Seven associations were statistically significant in both studies. We estimated a summary OR for these SNPs using a random effects meta-analysis applied to EGEA and the separate ECRHS countries (Figure 1). The OR estimates from the pooled analysis and random effects meta-analysis were similar. Forest plots showed highly consistent results in the different countries for the association between usual cough and *TRPV1 *rs17706630 (p heterogeneity = 0.932) and rs2277675 (p = 0.886) and consistency for the association between *TRPV1 *rs17706630 (p = 0.162), rs2277675 (p = 0.275), and rs224498 (p = 0.542) and nocturnal cough, whereas heterogeneity was observed for the association between two *TRPV1 *SNPs and nocturnal cough (rs161365; p = 0.048 and rs150854; p = 0.040). The associations between *TRPV1 *SNPs and cough symptoms did not differ between men and women (p for interaction > 0.05 for all SNPs). Further, excluding those with FEV1 < 80% predicted did not change the results.

**Table 3:** Association of *TRPV1 *SNPs with nocturnal cough, usual cough, and chronic cough under an additive model in 844 adults with asthma and 2046 adults without asthma

				Subjects with asthma			Subjects without asthma		
				
SNP	Region	Alleles^†^	MAF	Nocturnal cough	Usual cough	Chronic cough	Nocturnal cough	Usual cough	Chronic cough
rs4790522	3'UTR	C/A	0.41	0.97 (0.78-1.20)	**1.35 (1.09-1.67)**	1.19 (0.92-1.55)	**1.20 (1.03-1.39)**	**1.31 (1.10-1.57)**	1.01 (0.77-1.31)
rs16953163	intron	A/G	0.20	0.95 (0.73-1.23)	0.83 (0.64-1.09)	0.81 (0.58-1.12)	1.10 (0.92-1.30)	1.08 (0.88-1.34)	1.15 (0.84-1.57)
rs224546	intron	T/C	0.42	0.96 (0.77-1.18)	**1.27 (1.03-1.58)**	1.07 (0.83-1.39)	**1.16 (1.00-1.35)**	1.09 (0.91-1.30)	0.89 (0.68-1.15)
rs11655540	intron	T/G	0.35	1.03 (0.83-1.28)	0.89 (0.71-1.10)	1.03 (0.79-1.34)	0.77 (0.66-0.90) *	0.85 (0.70-1.03)	1.09 (0.83-1.43)
rs161364	intron	C/T	0.28	0.93 (0.73-1.17)	1.12 (0.89-1.42)	1.11 (0.84-1.47)	1.09 (0.93-1.29)	**1.22 (1.01-1.49)**	1.13 (0.84-1.52)
rs8065080	Ile585Val	T/C	0.39	0.94 (0.76-1.15)	0.94 (0.76-1.15)	0.91 (0.70-1.18)	1.15 (0.99-1.33)	0.92 (0.77-1.10)	0.90 (0.69-1.19)
rs161365	intron	C/T	0.32	1.11 (0.89-1.39)	0.91 (0.73-1.15)	0.86 (0.66-1.13)	0.72 (0.62-0.85)*^‡^	0.85 (0.70-1.05)	1.02 (0.76-1.38)
rs150908	intron	G/A	0.44	0.87 (0.71-1.07)	1.00 (0.81-1.23)	0.94 (0.73-1.20)	1.10 (0.95-1.28)	**1.23 (1.03-1.48)**	1.21 (0.93-1.58)
rs224534	Thr469Ile	G/A	0.35	0.86 (0.69-1.06)	0.90 (0.73-1.11)	0.97 (0.76-1.25)	1.01 (0.87-1.18)	1.14 (0.96-1.36)	1.24 (0.96-1.61)
rs17706630	intron	G/A	0.27	1.02 (0.81-1.30)	0.95 (0.75-1.21)	1.00 (0.76-1.32)	0.71 (0.59-0.84)*^‡^	**0.73 (0.59-0.92)**^‡^	**0.71 (0.50-0.99)**
rs222748	His167His	G/A	0.11	1.00 (0.72-1.40)	0.92 (0.66-1.28)	1.20 (0.82-1.76)	0.97 (0.76-1.23)	1.18 (0.89-1.56)	1.32 (0.88-1.97)
rs150846	intron	G/A	0.36	1.12 (0.91-1.38)	1.08 (0.87-1.33)	1.11 (0.85-1.44)	**1.22 (1.05-1.41)**	1.18 (0.99-1.42)	1.25 (0.96-1.62)
rs2277675	5'UTR	T/C	0.28	1.17 (0.93-1.46)	0.97 (0.77-1.21)	1.04 (0.79-1.35)	0.73 (0.62-0.86)*^‡^	0.64 (0.52-0.80)*^‡^	**0.64 (0.46-0.90)**
rs161381	5'UTR	G/T	0.15	1.05 (0.79-1.39)	1.21 (0.91-1.61)	1.06 (0.75-1.50)	1.20 (0.98-1.47)	1.19 (0.93-1.51)	1.07 (0.75-1.55)
rs222738	5'UTR	C/T	0.09	0.99 (0.69-1.40)	0.93 (0.64-1.33)	1.14 (0.73-1.77)	0.84 (0.65-1.09)	1.12 (0.82-1.51)	1.37 (0.89-2.09)
rs17707155	5'UTR	C/T	0.28	0.91 (0.73-1.14)	0.97 (0.77-1.21)	0.99 (0.75-1.31)	**1.23 (1.05-1.43)**	1.01 (0.83-1.22)	1.05 (0.78-1.40)
rs222741	5'	A/G	0.24	1.07 (0.85-1.34)	1.15 (0.90-1.46)	1.11 (0.83-1.50)	1.10 (0.93-1.30)	**1.23 (1.01-1.51)**	**1.40 (1.04-1.88)**
rs150854	5'	T/G	0.46	1.12 (0.91-1.37)	0.96 (0.78-1.18)	0.94 (0.73-1.21)	0.74 (0.64-0.86)*^‡^	0.84 (0.70-1.00)	**0.76 (0.57-1.00)**
rs224498	5'	T/G	0.39	0.93 (0.76-1.15)	1.16 (0.94-1.43)	0.99 (0.76-1.27)	1.32 (1.14-1.53)*^‡^	1.11 (0.92-1.33)	1.00 (0.76-1.31)

Results are presented as OR (95% CI) adjusted for age, sex, smoking habits, occupational exposure, principal components for within-Europe diversity, study, and sample

Bold type indicates *P *< 0.05, * *P*-value adjusted for multiple comparisons < 0.05. ^† ^Major/minor allele. MAF, minor allele frequency.^‡ ^*P *< 0.05, both in EGEA and ECRHS

In subjects with asthma, two *TRPV1 *SNPs were associated with usual cough, but the associations did not remain significant after correction for multiple testing (Table 3). Associations between SNPs in *TRPV4 *and *TRPA1 *and cough symptoms in subjects with or without asthma were also not significant after adjusting for multiple testing (Additional file 1: Tables S3 and S4).

### Haplotype-based association analysis *TRPV1 *and cough

Pairwise LD (r^2^) between the *TRPV1 *SNPs that were associated with cough symptoms was ≤ 0.72 (Additional file 1: Figure S3). We performed a sliding window approach to construct haplotypes across combinations of successive SNPs that were associated with nocturnal cough and usual cough in subjects without asthma. The best result for nocturnal cough was a 7-SNP haplotype comprising adjacent SNPs from rs8065080 to rs150846 (p-global = 4.8 × 10^-6^, Additional file 1: Figure S6). For usual cough, a 9-SNP haplotype comprising adjacent SNPs from rs17706630 to rs150854 showed the most significant association (p-global = 4.5 × 10^-6^, Additional file 1: Figure S7). Tables 4 and 5 show association analysis of specific *TRPV1 *haplotypes and nocturnal cough and usual cough, respectively. The haplotype analysis supported the results obtained by single SNP analysis.

**Table 4:** Haplotype-based association analysis of *TRPV1 *haplotypes and nocturnal cough in 2046 subjects without asthma

Haplotype	Haplotype frequency	OR (95%CI)	*P *value
TTGGAGG	0.227	1.00 (reference)	
CCAAGGG	0.131	1.26 (0.97-1.64)	0.085
CCGGGGA	0.120	1.54 (1.18-2.02)	0.001*
TCAAGGG	0.090	1.40 (1.04-1.90)	0.027
TCAGGGA	0.087	1.55 (1.14-2.10)	0.004*
CCGGGGG	0.079	1.51 (1.10-2.05)	0.010
TCAAGAA	0.061	1.53 (1.08-2.16)	0.016
Rare haplotypes	0.205	1.19 (0.93-1.53)	0.161

Haplotypes are a combination of 7 adjacent SNPs: rs8065080 T/C - rs161365 C/T - rs150908 G/A - rs224534 G/A - rs17706630 G/A - rs222748 G/A - rs150846 G/A. OR and 95% CI are adjusted for age, sex, smoking habits, occupational exposure, principal components for within-Europe diversity, study, and sample. *Significant after Bonferroni correction (0.05/7 = 0.007)

**Table 5:** Haplotype-based association analysis of *TRPV1 *haplotypes and usual cough in 2046 subjects without asthma

Haplotype	Haplotype frequency	OR (95%CI)	*P *value
AGGCGCCAG	0.224	1.00 (reference)	
GGGTGCTAT	0.158	1.25 (0.92-1.71)	0.152
GGGTGCCAG	0.140	1.53 (1.13-1.08)	0.006*
GGATTCCGT	0.123	1.54 (1.11-2.14)	0.010
GGATGCTAT	0.099	1.24 (0.87-1.77)	0.241
GAATGTCGT	0.070	1.60 (1.09-2.35)	0.016
Rare haplotypes	0.186	1.15 (0.84-1.58)	0.382

Haplotypes are a combination of 9 adjacent SNPs: rs17706630 G/A - rs222748 G/A - rs150846 G/A - rs2277675 T/C - rs161381 G/T - rs222738 C/T - rs17707155 C/T - rs222741 A/G - rs150854 T/G. OR and 95% CI are adjusted for age, sex, smoking habits, occupational exposure, principal components for within-Europe diversity, study, and sample. *Significant after Bonferroni correction (0.05/6 = 0.0083)

### Gene-environment interactions between *TRPV1 *polymorphisms and occupational exposure or smoking

We explored whether the effect of occupational exposure or current smoking on cough was enhanced by SNPs in *TRPV1 *because consistent main effects on cough symptoms were observed for this gene. In subjects without asthma, three *TRPV1 *SNPs appeared to modify the association between current smoking and cough symptoms (9.0 × 10^-4 ^≤ p interaction ≤ 0.030; Additional file 1: Table S5). In subjects with asthma, the association between occupational exposure and usual cough and chronic cough appeared to be modified by *TRPV1 *rs224498. Positive and significant associations between occupational exposure and cough were observed among TT subjects (OR 1.82 [1.11-2.99] for usual cough; OR 2.58 [1.39-4.79] for chronic cough), whereas ORs were close to unity and not statistically significant among GT subjects and GG subjects (p interaction = 0.006 for usual cough and p = 0.025 for chronic cough).

## Discussion

The present study showed significant associations between *TRPV1 *SNPs and cough symptoms among subjects without asthma from two independent European studies. Although we carried out a pooled analysis to increase the power of detecting associations of SNPs with cough symptoms, we verified that the results were similar in the EGEA and ECRHS studies, and reached the 5% significance level for five SNPs in each study. Random-effects meta-analysis of these SNPs showed similar estimates of the summary ORs with those obtained from the pooled analysis and no evidence of heterogeneity across the different studies and countries involved for three of these SNPs. Haplotype-based association analysis confirmed the single SNP analyses, and global p-values for association between *TRPV1 *haplotypes and cough were highly significant (p < 5 × 10^-6^).

The functional relevance of these *TRPV1 *SNPs, or variants not genotyped but in high LD with one of these SNPs, is so far unknown. *TRPV1 *variants could result in either activity or expression changes of TRPV1 channels in airway nerves or airways smooth muscle, influencing sensitivity for TRPV1-activating agents among carriers of such a variant. In a recent report, Cantero-Recasens et al.[5] have shown that a isoleucine-to-valine mutation at position 585 of the TRPV1 protein results in a 20-30% loss of channel function, and that the corresponding *TRPV1 *I585V (rs8065080) SNP is associated with a significantly lower risk of wheeze and cough in children with asthma. The structural explanation for the altered activity of TRPV1 585 V is not known at present, and channel activity of other *TRPV1 *variants needs to be evaluated. In the present analysis, *TRPV1 *I585V was also associated with a lower risk of nocturnal cough in EGEA adults with asthma (OR 0.62 [0.40-0.96], p = 0.03), but the association was not statistically significant in the pooled analysis. We did not find evidence of an association between common *TRPA1 *or *TRPV4 *variants and cough. The lack of association between SNPs in *TRPA1 *and cough was especially surprising, since many ubiquitous environmental irritants have been shown to activate TRPA1 receptors to cause cough [13,21]. The significant associations of *TRPV1 *variants with cough imply that our study population was large enough to reveal moderately increased or decreased risk estimates, suggesting that the lack of association cannot be attributed to a lack of power in our study. Our study was the first to explore associations of *TRPA1 *variants and respiratory outcomes. We cannot exclude the possibility that other respiratory outcomes such as bronchial hyperresponsiveness or more specific (irritant-induced) cough phenotypes are associated with these variants. Moreover, other unidentified rare genetic variants in *TRPA1 *or *TRPV4 *may play a role.

*TRPV1 *and *TRPA1 *are prime candidate genes for gene-environment interactions with exposures to a wide range of irritants and chemicals that may be encountered in the workplace, but also in air pollution and in cigarette smoke. The present findings seem to support this hypothesis by suggesting that interactions between *TRPV1 *SNPs and occupational exposures and smoking may modify the risk of cough symptoms. However, given the large number of tests performed, our findings on gene-environment interaction should be taken cautiously. Our observation that *TRPV1 *variants interact with irritants that may be present in occupational exposures or cigarette smoke to increase the risk of cough symptoms needs replication in other epidemiological or functional studies.

Chronic cough is one of the most frequent reasons for consultation with a primary care or respiratory physician, and may cause important adverse psychosocial and physical effects on patients' quality of life [36]. There is an unmet need for effective anti-tussive drugs for cough patients [37]. TRPV1 is being pursued as one of the potential therapeutic targets, and TRPV1 antagonists are being developed [13,37]. The results of our genetic association study seem to support the hypothesis that modulation of TRPV1 channel activity may provide therapeutic benefit in cough. However, some TRPV1 antagonists cause significant side effects on body temperature, which has necessitated the withdrawal of these compounds from clinical trials [13]. To date, both the scientific community and pharmaceutical industry are centered in the finding of a TRPV1 modulator to treat pain, without affecting temperature homeostasis. Potentially, the finding of such a modulator could be used as an antitussive agent.

Although cough is frequently associated with asthma [29], it has also been shown that in a general population, chronic cough may present as an independent symptom [38]. Further, recent observations of chronic cough in subjects without obvious respiratory disease such as asthma, have suggested the existence of a distinct clinical entity, the cough hypersensitivity syndrome [39,40]. The mechanisms of idiopathic cough are unclear, but the enhanced cough reflex in patients may result from increased sensitivity of cough receptors such as TRPV1 [39]. In non-asthmatic chronic cough patients, increased expression of TRPV1 was shown [3]. In our study, *TRPV1 *polymorphisms were associated with cough, but only in subjects without asthma. It is worth noting that associations with cough in those without asthma hold for those without airflow limitation, *i.e. *in subjects with cough independent of asthma or COPD. Thus, it could be hypothesized that genetic variation in *TRPV1 *increases the risk of an enhanced cough reflex among subjects without asthma. In asthmatics, the situation may be more complex, and the (modest) effect of genetic variation on TRPV1 channel activity may be obscured by the influence of other mechanims such as airway inflammation. For example, in asthma patients, chronic cough is associated with poor control of asthma and the use of inhaled corticosteroids [41]. To further elucidate the difference between subjects with and without asthma, studies with more specific cough phenotypes would be useful. Future replication studies could assess whether *TRPV1 *polymorphisms may be associated with greater cough sensitivity to inhaled capsaicin in cough patients (with and without asthma). Such a study could also include *TRPV1 *gene expression analysis before and after capsaicin challenge.

Women have greater cough sensitivity to inhaled capsaicin than men, as was shown in cough patients and healthy volunteers [42,43]. The reason for the greater sensitivity among women is unknown, and it is unlikely that smaller airway size and sex hormones explain these differences [42,43]. In the present study, cough prevalence was also strongly associated with female sex, in particular among asthmatics, but the associations between *TRPV1 *SNPs and cough symptoms were not different for men and women.

We used occupational exposure to vapors, gases, dust, and/or fumes as a proxy of work-related irritant exposure. VGDF comprises a wide variety of exposures to occupational agents, including irritants that may induce cough by activation of TRP receptors. One can assume that misclassification of irritant exposures by VGDF is of a non-differential nature, resulting in estimated measures of association that are biased toward the null, and a negative impact on the power to observe gene-environment interactions between VGDF exposure and *TRP *variants. Asthma was unrelated to VGDF exposure, a result consistent with previous results [24]. However, exposure to VGDF may modify the expression of asthma by increasing cough, which appears to be long-lasting since not all subjects were currently exposed. Long-term respiratory effects of occupational irritant exposures have been shown before, for example among women formerly employed in domestic cleaning [22].

## Conclusion

*TRPV1 *SNPs were associated with nocturnal, usual, and chronic cough in subjects without asthma from two independent studies in eight European countries. Irritant exposures such as cigarette smoking and occupational exposures were associated with cough symptoms. Exploratory findings on gene-environment interaction suggested that these associations may be enhanced by *TRPV1 *SNPs.

## Abbreviations

CI: Confidence interval; COPD: Chronic obstructive pulmonary disease; ECRHS: European Community Respiratory Health Survey; EGEA: Epidemiological study on the Genetics and Environment of Asthma; JEM: Job-exposure matrix; LD: Linkage disequilibrium; OR: Odds ratio; SNP: Single nucleotide polymorphism; TRPA: Transient receptor potential ankyrin; TRPV: Transient receptor potential vanilloid; VGDF: Vapors: gases: dusts: and/or fumes.

## Competing interests

The authors declare that they have no competing interests.

## Authors' contributions

LAMS, JMA, MAV, and FK were involved in the conception, hypotheses delineation, and design of the study. All the above authors and also EB, JRG, NLM, HK, AEC, IP, DJ, RV, CJ, JH, IG and ML participated in the acquisition of the data or the analysis and interpretation of such information. LAMS, MK and FK wrote the article. All authors reviewed and commented on the paper and JMA, JRG, AEC, RV, JH, EB, FD and MAV had substantial involvement in its revision prior to submission. All authors read and approved the final manuscript.

### EGEA cooperative group

Coordination: F Kauffmann; F Demenais (genetics); I Pin (clinical aspects). Respiratory epidemiology: Inserm U 700, Paris M Korobaeff (Egea1), F Neukirch (Egea1); Inserm U 707, Paris: I Annesi-Maesano; Inserm CESP/U 1018, Villejuif: F Kauffmann, N Le Moual, R Nadif, MP Oryszczyn; Inserm U 823, Grenoble: V Siroux Genetics: Inserm U 393, Paris: J Feingold; Inserm U 946, Paris: E Bouzigon, F Demenais, MH Dizier; CNG, Evry: I Gut, M Lathrop. Clinical centers: Grenoble: I Pin, C Pison; Lyon: D Ecochard (Egea1), F Gormand, Y Pacheco; Marseille: D Charpin (Egea1), D Vervloet; Montpellier: J Bousquet; Paris Cochin: A Lockhart (Egea1), R Matran (now in Lille); Paris Necker: E Paty, P Scheinmann; Paris-Trousseau: A Grimfeld, J Just. Data and quality management: Inserm ex-U155 (Egea1), Paris: J Hochez; Inserm CESP/U 1018, Villejuif: N Le Moual, Inserm ex-U780, Villejuif: C Ravault; Inserm ex-U794, Evry: N Chateigner; Grenoble: J Ferran.

### ECRHS, list of Principal Investigators and Senior Scientific Team (Members of the ECRHS Steering Committee in italics)

France: Paris (*F Neukirch, B Leynaert*, R Liard, M Zureik), Grenoble (I Pin, J Ferran-Quentin). Germany: Erfurt (*J Heinrich, M Wjst*, C Frye, I Meyer). Norway: Bergen (A. Gulsvik, E. Omenaas, *C. Svanes*, B. Laerum). Spain: Barcelona (*JM Antó*, J Sunyer, M Kogevinas, JP Zock, × Basagana, F Burgos), Huelva (J Maldonado, A Pereira, JL Sanchez), Albacete (J Martinez-Moratalla Rovira, E Almar), Galdakao (N Muniozguren, I Urritia), Oviedo (F Payo). Sweden: Uppsala (*C Janson*, G Boman, D Norback, M Gunnbjornsdottir), Umea (E Norrman, M Soderberg, K Franklin, B Lundback, B Forsberg, L Nystrom). Switzerland: Basel (*N Künzli*, B Dibbert, M Hazenkamp, M Brutsche, *U Ackermann-Liebrich)*. United Kingdom: *P Burney, S Chinn, D Jarvis*, Norwich (D Jarvis, B Harrison), Ipswich (D Jarvis, R Hall, D Seaton).

## Supplementary Material

Additional file 1 **Additional Methods, Tables and Figures**. Click here for file
