# Supplementary material for: Transient receptor potential genes, smoking, occupational exposures and cough in adults
Source: Respir Res. 2012 Mar 23;13(1):26. doi: 10.1186/1465-9921-13-26 (PMC3342106; doi:10.1186/1465-9921-13-26)
Supplement: Additional file 1 — Additional Methods, Tables and Figures. [file 1465-9921-13-26-S1.DOC]

**ADDITIONAL FILE**

**Transient receptor potential genes, smoking, occupational exposures and cough in adults**

Lidwien A.M. Smit, Manolis Kogevinas, Josep M. Antó, Emmanuelle Bouzigon, Juan Ramón González, Nicole Le Moual, Hans Kromhout, Anne-Elie Carsin, Isabelle Pin, Deborah Jarvis, Roel Vermeulen, Christer Janson, Joachim Heinrich, Ivo Gut, Mark Lathrop, Miguel A. Valverde, Florence Demenais, Francine Kauffmann

**Asthma**

In EGEA, inclusion criteria used to define (ever) asthma in probands were based on self-reported positive answers to the four questions “Have you ever had attacks of breathlessness at rest with wheezing?”, “Have you ever had asthma attacks?”, “Was this diagnosis confirmed by a physician?”, and “Have you had an asthma attack in the last 12 months?”, or a positive response to at least two questions and a positive review of their medical record [E1]. Asthma in relatives of probands was defined as a positive answer to at least one of the first two questions [E1]. In ECRHS, asthma was defined as a positive answer to the question “Have you ever had asthma?”.

**Occupational exposure**

Subjects reported their two most recent jobs in EGEA, and their complete job history in ECRHS. EGEA subjects who had a job history of more than two jobs, answered a question on exposure to VGDF in earlier jobs. Only 4 EGEA subjects (0.6%) who were classified by the JEM as unexposed in their two most recent jobs answered positively to this question. In total, 72 ECRHS subjects (3.3%) were classified by the JEM as unexposed in their two most recent jobs, but were exposed to VGDF in earlier jobs. Results did not change when these subjects were classified as exposed. The significant association between chronic cough and occupational exposure disappeared when we excluded individuals who were more than one year ago employed in a job with exposure to VGDF, but who were not currently exposed (OR 1.15 [0.79-1.68]).

**Genotyping**

Selected SNPs fulfilled the quality control (QC) criteria that have been applied to the Gabriel genome-wide study [E2]. The QC criteria for SNPs included call rate >=97%, minor allele frequency >=1%, and Hardy-Weinberg (HW) *P*-value >10^-4^ [E2].

**Statistical analysis**

All subjects were genetically unrelated, but the EGEA sample included 246 pairs of spouses. To account for dependence among spouses, we estimated odds ratios (ORs) and 95% confidence intervals (CIs) by generalized estimating equations (GEE models) using household as a cluster. We assumed an exchangeable correlation structure with a common correlation for each pair of spouses. The effect of single *TRP* SNPs on disease was tested under an additive genetic model with the minor allele as risk allele. Population admixture was taken into account by including informative principal components for within-Europe diversity for EGEA and ECRHS as covariates in the association analysis [E2]. Age, smoking, sex, occupational exposure, study and sample (dummy indicators for EGEA, random sample ECRHS, and symptomatic sample of ECRHS) were also incorporated in the logistic model. *P*-values of association were corrected to address multiple testing (n=174 tests, i.e. 58 SNPs x 3 cough outcomes) by using the Benjamini-Hochberg method [E3]. This is a heuristic method that is robust against positive dependence among *P*-values (e.g. correlation between SNPs or phenotypes). Haplotype association analyses were assessed using SNPassoc [E4] and haplo.stats [E5] R packages. To refine the haplotype construction and to identify a potential causative core region, we further used an unbiased sliding window approach to construct successive and adjacent 2- to 9-SNPs haplotypes across combination of successive SNPs that were statistically significantly associated with the phenotype. Furthermore, a permutation procedure was used to estimate the significance of the best result (based on 1000 permutations) and to avoid the multiple comparison problem. The haplotype association test was carried out by looking at the *P*-value obtained from a global test. This global test was performed by using a likelihood ratio test and then a score test for a given haplotype configuration was performed if the global test was significant. Haplotypes with frequencies lower than 5% were combined into a single category (rare haplotypes). To explore whether the effect of occupational exposure or current smoking on cough was modified by *TRP* genotype we introduced a multiplicative gene-environment interaction term in the GEE model (SNP x occupational exposure or SNP x current smoking). The statistical significance of the interaction term was assessed by using a generalized score test which follows a chi-square distribution with 1 degree of freedom [E6].

**Table E1.** Association of *TRPV1* SNPs with nocturnal cough, usual cough, and chronic cough under an additive model in 203 EGEA adults with asthma and 486 EGEA adults without asthma.

|  |  |  |  | Subjects with asthma | | |  | Subjects without asthma | | |
| --- | --- | --- | --- | --- | --- | --- | --- | --- | --- | --- |
| SNP | Region | Alleles^†^ | MAF | Nocturnal cough | Usual cough | Chronic cough |  | Nocturnal cough | Usual cough | Chronic cough |
| rs4790522 | 3’UTR | C/A | 0.41 | 1.16 (0.74-1.82) | 1.36 (0.85-2.16) | 1.32 (0.77-2.27) |  | 1.13 (0.80-1.59) | 1.29 (0.89-1.86) | 1.11 (0.73-1.69) |
| rs16953163 | intron | A/G | 0.20 | 0.92 (0.55-1.53) | 0.70 (0.40-1.24) | 0.76 (0.41-1.39) |  | 1.40 (0.95-2.06) | 0.98 (0.60-1.62) | 0.79 (0.39-1.60) |
| rs224546 | intron | T/C | 0.42 | 1.02 (0.66-1.59) | 1.15 (0.73-1.83) | 1.17 (0.67-2.04) |  | 1.10 (0.78-1.55) | 0.99 (0.65-1.52) | 1.00 (0.55-1.82) |
| rs11655540 | intron | T/G | 0.35 | 1.01 (0.63-1.62) | 0.92 (0.57-1.49) | 0.85 (0.50-1.46) |  | 0.71 (0.50-1.03) | 0.91 (0.58-1.44) | 1.14 (0.62-2.10) |
| rs161364 | intron | C/T | 0.28 | **1.65 (1.00-2.71)** | 1.14 (0.71-1.80) | 1.22 (0.70-2.15) |  | 0.82 (0.55-1.21) | 0.92 (0.58-1.45) | 0.80 (0.38-1.68) |
| rs8065080 | Ile585Val | T/C | 0.39 | **0.62 (0.40-0.96)** | 0.96 (0.62-1.47) | 0.96 (0.59-1.57) |  | **1.64 (1.18-2.28)** | 1.02 (0.67-1.55) | 1.20 (0.63-2.28) |
| rs161365 | intron | C/T | 0.32 | 0.97 (0.59-1.59) | 0.84 (0.51-1.39) | 0.70 (0.39-1.24) |  | **0.60 (0.40-0.90)**^‡^ | 0.97 (0.62-1.53) | 1.10 (0.60-2.04) |
| rs150908 | intron | G/A | 0.44 | 1.28 (0.81-2.03) | 1.09 (0.69-1.73) | 1.05 (0.61-1.81) |  | 1.21 (0.87-1.70) | 1.05 (0.71-1.54) | 0.96 (0.54-1.69) |
| rs224534 | Thr469Ile | G/A | 0.35 | 0.97 (0.62-1.53) | 1.04 (0.65-1.66) | 1.13 (0.64-1.98) |  | 1.34 (0.95-1.87) | 1.17 (0.81-1.69) | 1.07 (0.68-1.70) |
| rs17706630 | intron | G/A | 0.27 | 1.06 (0.62-1.81) | 0.80 (0.47-1.36) | 0.74 (0.41-1.33) |  | **0.45 (0.27-0.77)**^‡^ | **0.56 (0.32-0.98)**^‡^ | 0.45 (0.20-1.00) |
| rs222748 | His167His | G/A | 0.11 | 0.81 (0.40-1.66) | 1.21 (0.58-2.52) | 1.24 (0.54-2.83) |  | 1.40 (0.84-2.35) | 1.41 (0.73-2.71) | 1.31 (0.50-3.41) |
| rs150846 | intron | G/A | 0.36 | 1.09 (0.70-1.72) | 1.19 (0.73-1.95) | 1.33 (0.76-2.34) |  | 1.19 (0.85-1.66) | 1.30 (0.89-1.90) | 1.36 (0.90-2.04) |
| rs2277675 | 5’UTR | T/C | 0.28 | 1.26 (0.78-2.03) | 1.01 (0.64-1.62) | 0.91 (0.54-1.54) |  | **0.55 (0.35-0.87)**^‡^ | **0.48 (0.28-0.83)**^‡^ | **0.24 (0.09-0.62)** |
| rs161381 | 5’UTR | G/T | 0.15 | 1.60 (0.85-3.00) | 0.93 (0.52-1.67) | 0.86 (0.45-1.67) |  | 0.97 (0.64-1.47) | 1.18 (0.73-1.90) | 1.18 (0.59-2.38) |
| rs222738 | 5’UTR | C/T | 0.09 | 0.95 (0.44-2.06) | 0.94 (0.42-2.13) | 0.81 (0.31-2.16) |  | 0.87 (0.49-1.55) | 0.75 (0.33-1.69) | 0.87 (0.25-3.05) |
| rs17707155 | 5’UTR | C/T | 0.28 | 0.71 (0.44-1.15) | 0.84 (0.51-1.39) | 1.01 (0.57-1.80) |  | **1.48 (1.05-2.08)** | 1.17 (0.76-1.80) | 1.35 (0.69-2.62) |
| rs222741 | 5’ | A/G | 0.24 | 1.37 (0.80-2.32) | 0.90 (0.51-1.60) | 0.77 (0.40-1.50) |  | 1.02 (0.72-1.46) | 1.21 (0.78-1.90) | 1.75 (0.90-3.37) |
| rs150854 | 5’ | T/G | 0.46 | 1.13 (0.72-1.75) | 1.17 (0.74-1.85) | 1.17 (0.70-1.94) |  | **0.58 (0.41-0.83)**^‡^ | 0.70 (0.46-1.08) | **0.46 (0.24-0.87)** |
| rs224498 | 5’ | T/G | 0.39 | 1.03 (0.66-1.61) | 0.88 (0.56-1.40) | 0.85 (0.51-1.40) |  | **1.46 (1.04-2.04)**^‡^ | 1.21 (0.81-1.81) | 1.43 (0.82-2.51) |

Results are presented as OR (95% CI) adjusted for age, sex, smoking habits, occupational exposure, and principal components for within-Europe diversity.

Bold type indicates *P*<0.05, ^†^ Major/minor allele. MAF, minor allele frequency.^‡^ *P*<0.05, both in EGEA and ECRHS.

**Table E2.** Association of *TRPV1* SNPs with nocturnal cough, usual cough, and chronic cough under an additive model in 641 ECRHS adults with asthma and 1560 ECRHS adults without asthma.

|  |  |  |  | Subjects with asthma | | |  | Subjects without asthma | | |
| --- | --- | --- | --- | --- | --- | --- | --- | --- | --- | --- |
| SNP | Region | Alleles^†^ | MAF | Nocturnal cough | Usual cough | Chronic cough |  | Nocturnal cough | Usual cough | Chronic cough |
| rs4790522 | 3’UTR | C/A | 0.41 | 0.91 (0.71-1.16) | **1.37 (1.06-1.76)** | 1.16 (0.85-1.59) |  | **1.22 (1.03-1.44)** | **1.34 (1.09-1.63)** | 0.99 (0.72-1.36) |
| rs16953163 | intron | A/G | 0.19 | 0.95 (0.71-1.28) | 0.84 (0.61-1.14) | 0.83 (0.56-1.22) |  | 1.04 (0.86-1.26) | 1.09 (0.86-1.38) | 1.26 (0.90-1.78) |
| rs224546 | intron | T/C | 0.42 | 0.93 (0.73-1.18) | **1.34 (1.05-1.72)** | 1.05 (0.78-1.42) |  | **1.18 (1.00-1.39)** | 1.13 (0.93-1.37) | 0.87 (0.65-1.17) |
| rs11655540 | intron | T/G | 0.35 | 1.04 (0.81-1.33) | 0.88 (0.68-1.13) | 1.09 (0.80-1.50) |  | **0.78 (0.66-0.93)** | 0.82 (0.66-1.02) | 1.06 (0.78-1.44) |
| rs161364 | intron | C/T | 0.28 | 0.76 (0.58-1.00) | 1.14 (0.87-1.49) | 1.08 (0.77-1.51) |  | 1.17 (0.97-1.40) | **1.33 (1.07-1.64)** | 1.25 (0.90-1.72) |
| rs8065080 | Ile585Val | T/C | 0.39 | 1.08 (0.85-1.37) | 0.92 (0.72-1.17) | 0.90 (0.66-1.23) |  | 1.06 (0.90-1.25) | 0.89 (0.73-1.09) | 0.84 (0.61-1.14) |
| rs161365 | intron | C/T | 0.32 | 1.16 (0.90-1.50) | 0.95 (0.73-1.22) | 0.95 (0.69-1.29) |  | **0.75 (0.63-0.90)**^‡^ | 0.83 (0.66-1.04) | 0.99 (0.70-1.39) |
| rs150908 | intron | G/A | 0.44 | **0.77 (0.61-0.98)** | 0.99 (0.78-1.25) | 0.94 (0.70-1.27) |  | 1.08 (0.91-1.27) | **1.29 (1.06-1.58)** | 1.30 (0.96-1.76) |
| rs224534 | Thr469Ile | G/A | 0.35 | 0.82 (0.64-1.04) | 0.87 (0.68-1.10) | 0.96 (0.71-1.29) |  | 0.94 (0.80-1.12) | 1.14 (0.93-1.39) | 1.31 (0.96-1.78) |
| rs17706630 | intron | G/A | 0.27 | 1.03 (0.78-1.35) | 1.00 (0.76-1.32) | 1.10 (0.79-1.52) |  | **0.76 (0.63-0.92)**^‡^ | **0.77 (0.60-0.98)**^‡^ | 0.78 (0.53-1.13) |
| rs222748 | His167His | G/A | 0.11 | 1.06 (0.72-1.54) | 0.88 (0.60-1.29) | 1.23 (0.78-1.93) |  | 0.89 (0.68-1.17) | 1.13 (0.82-1.56) | 1.40 (0.90-2.20) |
| rs150846 | intron | G/A | 0.36 | 1.14 (0.90-1.44) | 1.08 (0.85-1.38) | 1.06 (0.79-1.43) |  | **1.23 (1.05-1.45)** | 1.17 (0.95-1.44) | 1.24 (0.91-1.71) |
| rs2277675 | 5’UTR | T/C | 0.28 | 1.14 (0.88-1.48) | 0.93 (0.72-1.21) | 1.03 (0.75-1.41) |  | **0.76 (0.64-0.92)**^‡^ | **0.67 (0.53-0.85)**^‡^ | 0.76 (0.53-1.09) |
| rs161381 | 5’UTR | G/T | 0.15 | 0.93 (0.67-1.29) | 1.33 (0.96-1.84) | 1.12 (0.75-1.68) |  | **1.28 (1.02-1.61)** | 1.20 (0.91-1.59) | 1.00 (0.65-1.56) |
| rs222738 | 5’UTR | C/T | 0.09 | 1.00 (0.67-1.48) | 0.93 (0.62-1.40) | 1.31 (0.78-2.19) |  | 0.84 (0.63-1.12) | 1.21 (0.86-1.68) | 1.55 (0.97-2.46) |
| rs17707155 | 5’UTR | C/T | 0.28 | 0.99 (0.77-1.28) | 1.00 (0.77-1.30) | 0.98 (0.72-1.35) |  | 1.17 (0.98-1.39) | 0.97 (0.78-1.20) | 0.98 (0.70-1.37) |
| rs222741 | 5’ | A/G | 0.24 | 1.01 (0.78-1.31) | 1.25 (0.95-1.63) | 1.27 (0.90-1.78) |  | 1.12 (0.93-1.35) | 1.24 (1.00-1.55) | 1.33 (0.95-1.85) |
| rs150854 | 5’ | T/G | 0.46 | 1.11 (0.87-1.40) | 0.90 (0.71-1.14) | 0.85 (0.63-1.15) |  | **0.78 (0.66-0.92)**^‡^ | 0.87 (0.71-1.07) | 0.84 (0.62-1.15) |
| rs224498 | 5’ | T/G | 0.39 | 0.91 (0.72-1.15) | 1.26 (0.99-1.60) | 1.03 (0.76-1.39) |  | **1.29 (1.10-1.52)**^‡^ | 1.09 (0.88-1.33) | 0.91 (0.66-1.25) |

Results are presented as OR (95% CI) adjusted for age, sex, smoking habits, occupational exposure, principal components for within-Europe diversity, and sample.

Bold type indicates *P*<0.05, ^†^ Major/minor allele. MAF, minor allele frequency.^‡^ *P*<0.05, both in EGEA and ECRHS.

**Table E3.** Association of *TRPV4* SNPs with nocturnal cough, usual cough, and chronic cough under an additive model in 844 adults with asthma and 2046 adults without asthma.

|  |  |  |  | Subjects with asthma | | |  | Subjects without asthma | | |
| --- | --- | --- | --- | --- | --- | --- | --- | --- | --- | --- |
| SNP | Region | Alleles^†^ | MAF | Nocturnal cough | Usual cough | Chronic cough |  | Nocturnal cough | Usual cough | Chronic cough |
| rs1861810 | intron | C/A | 0.41 | 0.87 (0.71-1.08) | 0.96 (0.78-1.20) | 0.89 (0.69-1.15) |  | 1.00 (0.86-1.16) | 1.08 (0.91-1.29) | 0.97 (0.75-1.25) |
| rs3742037 | Ile678Ile | G/A | 0.13 | 1.16 (0.87-1.54) | 0.87 (0.65-1.17) | 0.95 (0.67-1.35) |  | 1.20 (0.97-1.48) | 0.97 (0.74-1.26) | 1.11 (0.76-1.61) |
| rs10735104 | intron | T/C | 0.49 | 1.07 (0.87-1.30) | **1.26 (1.03-1.56)** | **1.36 (1.06-1.75)** |  | 0.98 (0.85-1.13) | 0.97 (0.82-1.15) | 0.93 (0.73-1.18) |
| rs12579553 | intron | A/G | 0.09 | 0.71 (0.50-1.02) | 0.94 (0.66-1.35) | 0.87 (0.56-1.36) |  | 0.90 (0.69-1.17) | 0.86 (0.63-1.19) | 0.98 (0.61-1.59) |
| rs10850783 | intron | C/A | 0.48 | 1.06 (0.86-1.29) | 1.13 (0.92-1.40) | 1.12 (0.87-1.45) |  | 1.06 (0.92-1.23) | 0.92 (0.77-1.10) | 0.95 (0.74-1.23) |
| rs1861809 | intron | G/A | 0.49 | 1.08 (0.88-1.32) | 1.15 (0.93-1.42) | 1.14 (0.89-1.47) |  | 1.08 (0.93-1.24) | 0.90 (0.76-1.08) | 0.91 (0.70-1.17) |
| rs10850800 | intron | A/G | 0.28 | 1.03 (0.82-1.30) | 0.90 (0.71-1.14) | 0.98 (0.73-1.30) |  | 0.91 (0.77-1.07) | 1.22 (1.00-1.48) | 1.04 (0.77-1.40) |
| rs10850830 | 5’UTR | C/T | 0.48 | 0.96 (0.78-1.18) | 0.86 (0.69-1.06) | 0.90 (0.69-1.17) |  | 1.08 (0.93-1.24) | 0.94 (0.78-1.12) | 1.09 (0.83-1.42) |
| rs10850838 | 5’UTR | C/T | 0.31 | 1.08 (0.86-1.35) | 1.01 (0.81-1.26) | 1.06 (0.82-1.39) |  | 1.14 (0.97-1.33) | 0.94 (0.77-1.14) | 1.02 (0.77-1.36) |
| rs6606743 | 5’UTR | G/A | 0.48 | 1.08 (0.88-1.33) | **0.79 (0.64-0.97)** | 0.80 (0.61-1.05) |  | 1.11 (0.97-1.28) | 1.02 (0.86-1.22) | 0.95 (0.73-1.23) |

Results are presented as OR (95% CI) adjusted for age, sex, smoking habits, occupational exposure, principal components for within-Europe diversity, study, and sample.

Bold type indicates P<0.05. ^†^ Major/minor allele. MAF, minor allele frequency.

**Table E4.** Association of *TRPA1* SNPs with nocturnal cough, usual cough, and chronic cough under an additive model in 844 adults with asthma and 2046 adults without asthma.

|  |  |  |  | Subjects with asthma | | |  | Subjects without asthma | | |
| --- | --- | --- | --- | --- | --- | --- | --- | --- | --- | --- |
| SNP | Region | Alleles^†^ | MAF | Nocturnal cough | Usual cough | Chronic cough |  | Nocturnal cough | Usual cough | Chronic cough |
| rs894886 | 3’UTR | C/T | 0.26 | 0.91 (0.73-1.15) | 0.91 (0.72-1.16) | 0.92 (0.69-1.23) |  | 1.03 (0.87-1.22) | 0.95 (0.77-1.17) | 0.79 (0.57-1.09) |
| rs12540984 | 3’UTR | A/G | 0.14 | 1.13 (0.84-1.5) | 1.12 (0.83-1.49) | 1.22 (0.85-1.74) |  | 1.13 (0.92-1.39) | **1.35 (1.05-1.72)** | **1.45 (1.01-2.07)** |
| rs4738201 | 3’UTR | A/G | 0.50 | 0.93 (0.76-1.15) | 1.07 (0.87-1.31) | 0.98 (0.76-1.26) |  | 1.01 (0.88-1.17) | 1.01 (0.84-1.20) | 1.02 (0.77-1.34) |
| rs6996723 | 3’UTR | C/T | 0.19 | **0.74 (0.57-0.95)** | 0.89 (0.68-1.16) | 0.82 (0.59-1.13) |  | 0.98 (0.81-1.18) | 0.98 (0.77-1.24) | 1.01 (0.70-1.45) |
| rs7827617 | 3’UTR | A/G | 0.17 | 1.26 (0.96-1.64) | 1.07 (0.82-1.40) | 1.13 (0.82-1.55) |  | 0.97 (0.80-1.17) | 0.87 (0.69-1.10) | 0.79 (0.54-1.15) |
| rs959974 | intron | G/T | 0.46 | 1.22 (1.00-1.50) | 1.03 (0.84-1.27) | 1.00 (0.78-1.30) |  | 0.98 (0.85-1.13) | 0.97 (0.82-1.16) | 1.07 (0.82-1.39) |
| rs959976 | His1018Arg | T/C | 0.17 | 1.25 (0.95-1.64) | 1.08 (0.83-1.41) | 1.18 (0.87-1.62) |  | 0.96 (0.79-1.16) | 0.87 (0.69-1.10) | 0.85 (0.59-1.23) |
| rs1384001 | intron | C/A | 0.46 | 1.22 (1.00-1.50) | 1.03 (0.84-1.27) | 1.00 (0.78-1.30) |  | 0.98 (0.85-1.13) | 0.97 (0.82-1.16) | 1.07 (0.82-1.39) |
| rs4738202 | intron | G/A | 0.31 | 0.89 (0.72-1.10) | 0.89 (0.72-1.11) | 0.88 (0.67-1.17) |  | 1.04 (0.89-1.21) | 1.01 (0.84-1.22) | 0.88 (0.66-1.19) |
| rs13280644 | Leu830Leu | C/T | 0.11 | 0.78 (0.56-1.08) | 1.10 (0.79-1.52) | 0.90 (0.60-1.35) |  | 0.90 (0.71-1.15) | 0.93 (0.68-1.27) | 1.00 (0.63-1.60) |
| rs13249568 | intron | T/C | 0.27 | 0.91 (0.72-1.15) | 0.91 (0.71-1.16) | 0.92 (0.68-1.24) |  | 0.98 (0.83-1.15) | 0.94 (0.76-1.16) | 0.86 (0.62-1.19) |
| rs10504523 | intron | G/A | 0.27 | 0.91 (0.72-1.15) | 0.90 (0.71-1.15) | 0.92 (0.68-1.24) |  | 0.98 (0.83-1.15) | 0.94 (0.77-1.16) | 0.86 (0.62-1.19) |
| rs1025926 | intron | C/T | 0.24 | 1.18 (0.92-1.51) | 1.09 (0.85-1.40) | 0.91 (0.67-1.25) |  | 1.00 (0.84-1.18) | 1.03 (0.85-1.26) | 1.21 (0.90-1.63) |
| rs10504524 | intron | G/T | 0.27 | 0.90 (0.71-1.14) | 0.89 (0.70-1.14) | 0.88 (0.65-1.19) |  | 0.98 (0.84-1.16) | 0.96 (0.78-1.18) | 0.85 (0.62-1.18) |
| rs1025927 | intron | A/G | 0.10 | 0.73 (0.52-1.03) | 1.08 (0.76-1.53) | 0.82 (0.54-1.25) |  | 0.93 (0.72-1.19) | 0.89 (0.64-1.24) | 1.13 (0.70-1.84) |
| rs1025928 | intron | C/T | 0.41 | 0.94 (0.77-1.15) | 0.99 (0.80-1.21) | 1.02 (0.79-1.31) |  | 0.98 (0.85-1.14) | 0.96 (0.80-1.14) | 0.87 (0.66-1.16) |
| rs10504525 | intron | C/T | 0.16 | 1.05 (0.79-1.39) | 0.79 (0.58-1.06) | 0.97 (0.68-1.40) |  | 1.04 (0.86-1.26) | 0.97 (0.76-1.23) | 0.81 (0.55-1.18) |
| rs3735943 | Leu544Leu | G/A | 0.48 | 1.17 (0.95-1.44) | 0.96 (0.78-1.18) | 1.03 (0.80-1.33) |  | 1.07 (0.92-1.23) | 1.08 (0.91-1.28) | 1.16 (0.88-1.52) |
| rs10504526 | intron | A/G | 0.48 | 1.16 (0.94-1.43) | 0.96 (0.78-1.18) | 1.03 (0.79-1.33) |  | 1.06 (0.92-1.22) | 1.07 (0.90-1.27) | 1.15 (0.87-1.50) |
| rs10109581 | intron | G/T | 0.27 | 1.08 (0.86-1.36) | 0.92 (0.73-1.17) | 1.06 (0.80-1.41) |  | 0.97 (0.83-1.14) | 0.90 (0.74-1.10) | 0.85 (0.62-1.16) |
| rs3735945 | intron | C/T | 0.11 | 1.11 (0.80-1.53) | 1.19 (0.86-1.65) | 1.20 (0.81-1.79) |  | 0.90 (0.71-1.13) | 0.84 (0.63-1.13) | 0.97 (0.63-1.48) |
| rs1443952 | intron | C/T | 0.32 | 1.17 (0.94-1.46) | 1.11 (0.89-1.39) | 1.06 (0.80-1.40) |  | 1.03 (0.89-1.21) | 1.11 (0.93-1.34) | 1.31 (0.98-1.75) |
| rs7010969 | intron | C/A | 0.41 | 0.81 (0.66-1.00) | 0.97 (0.78-1.19) | 0.90 (0.69-1.17) |  | 0.99 (0.85-1.14) | 1.00 (0.84-1.19) | 0.90 (0.68-1.19) |
| rs7011431 | intron | G/A | 0.27 | 1.08 (0.86-1.36) | 0.92 (0.73-1.17) | 1.06 (0.80-1.41) |  | 0.98 (0.83-1.14) | 0.90 (0.74-1.10) | 0.85 (0.62-1.17) |
| rs4738206 | intron | T/G | 0.32 | 1.16 (0.93-1.45) | 1.12 (0.90-1.40) | 1.06 (0.80-1.40) |  | 1.04 (0.89-1.21) | 1.10 (0.92-1.32) | 1.29 (0.97-1.72) |
| rs2278655 | intron | C/T | 0.08 | 1.03 (0.72-1.49) | 1.06 (0.73-1.54) | 1.29 (0.82-2.03) |  | 1.19 (0.91-1.55) | 1.04 (0.75-1.44) | 1.19 (0.74-1.92) |
| rs13268757 | Arg3Cys | G/A | 0.16 | 1.06 (0.79-1.41) | 0.80 (0.59-1.08) | 0.99 (0.69-1.43) |  | 1.04 (0.86-1.27) | 0.95 (0.75-1.22) | 0.75 (0.50-1.12) |
| rs2587567 | 5’UTR | T/C | 0.40 | 0.95 (0.77-1.16) | 1.02 (0.83-1.26) | 0.92 (0.71-1.20) |  | **0.80 (0.69-0.93)** | 0.86 (0.72-1.04) | 0.85 (0.64-1.12) |
| rs10093536 | 5’UTR | A/G | 0.21 | 1.25 (0.98-1.59) | 1.07 (0.83-1.36) | 1.20 (0.90-1.61) |  | **1.20 (1.01-1.43)** | 0.98 (0.79-1.23) | 0.95 (0.67-1.34) |

Results are presented as OR (95% CI) adjusted for age, sex, smoking habits, occupational exposure, principal components for within-Europe diversity, study, and sample. Bold type indicates P<0.05. ^†^ Major/minor allele. MAF, minor allele frequency.

**Table E5**. Associations between current smoking and cough symptoms in 2046 adults without asthma, stratified by *TRPV1* genotype

| SNP | Region | Alleles^†^ | Cough symptom | OR (95% CI) in subjects homozygous for major allele | OR (95% CI) in heterozygous subjects | OR (95% CI) in subjects homozygous for minor allele | *P* interaction  SNP x current smoking |
| --- | --- | --- | --- | --- | --- | --- | --- |
| rs150908 | intron | G/A | Usual cough | 5.56 (3.32-9.31) | 3.17 (2.24-4.49) | 2.56 (1.45-4.51) | 0.030 |
|  |  |  | Chronic cough | 8.65 (3.54-21.14) | 3.86 (2.27-6.57) | 1.87 (0.76-4.61) | 0.013 |
| rs224534 | Thr469Ile | G/A | Chronic cough | 8.99 (4.06-19.90) | 3.34 (1.98-5.64) | ne | 9.0x10^-4^ |
| rs2277675 | 5’UTR | T/C | Chronic cough | 2.99 (1.81-4.92) | 5.41 (2.52-11.60) | ne | 0.010 |

Results are presented as associations between current smoking (current versus never+ex smokers) and cough, adjusted for age, sex, occupational exposure, principal components for within-Europe diversity, study, and sample. ^†^ Major/minor allele. Ne: not estimated due to small numbers.

**Family members of asthmatic probands**

Parents and siblings

or

Spouse and children

**Family members of asthmatic probands**

Parents and siblings

or

Spouse and children

**Figure E1.** Flowchart of EGEA subjects.

Figure E1b. Flowchart of ECRHS subjetcts

**ECRHS II, second survey around 2000**

Follow-up, n=9175

**Present analysis**

Genotyping of asthma cases and sample of controls (8 countries with DNA)

**Asthma**

n=641

**No asthma**

n=1560

**ECRHS I, first survey around 1990**

20% Random sample, and subjects with asthma symptoms not included in random sample, 20-44 years, 12 countries, n=15,716

**Figure E2.** Flowchart of ECRHS subjects.

**
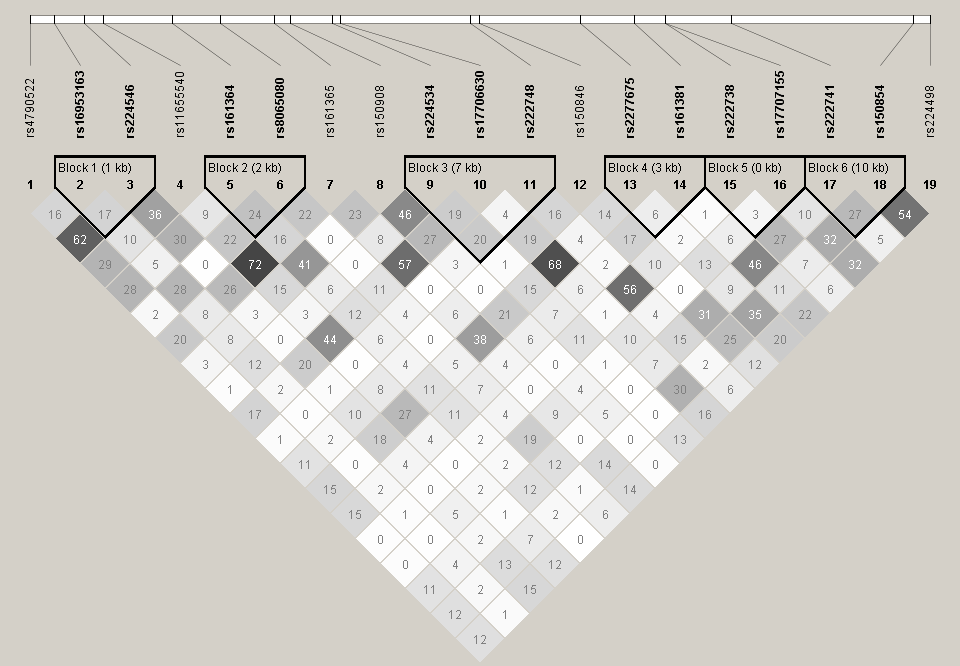
**

**Figure E3.** Linkage disequilibrium between 19 SNPs in *TRPV1* using the Haploview program [E7]. Values of r^2^ (x100) are shown.

**
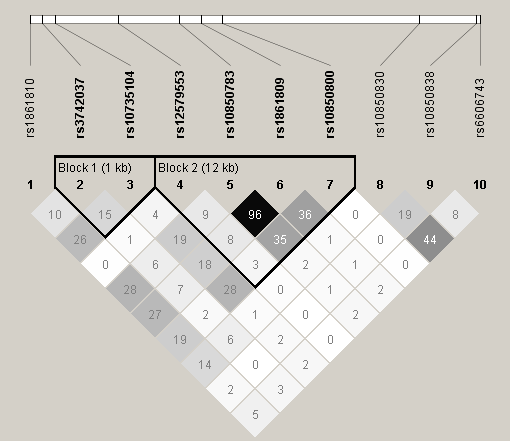
**

**Figure E4.** Linkage disequilibrium between 10 SNPs in *TRPV4* using the Haploview program [E7]. Values of r^2^ (x100) are shown.


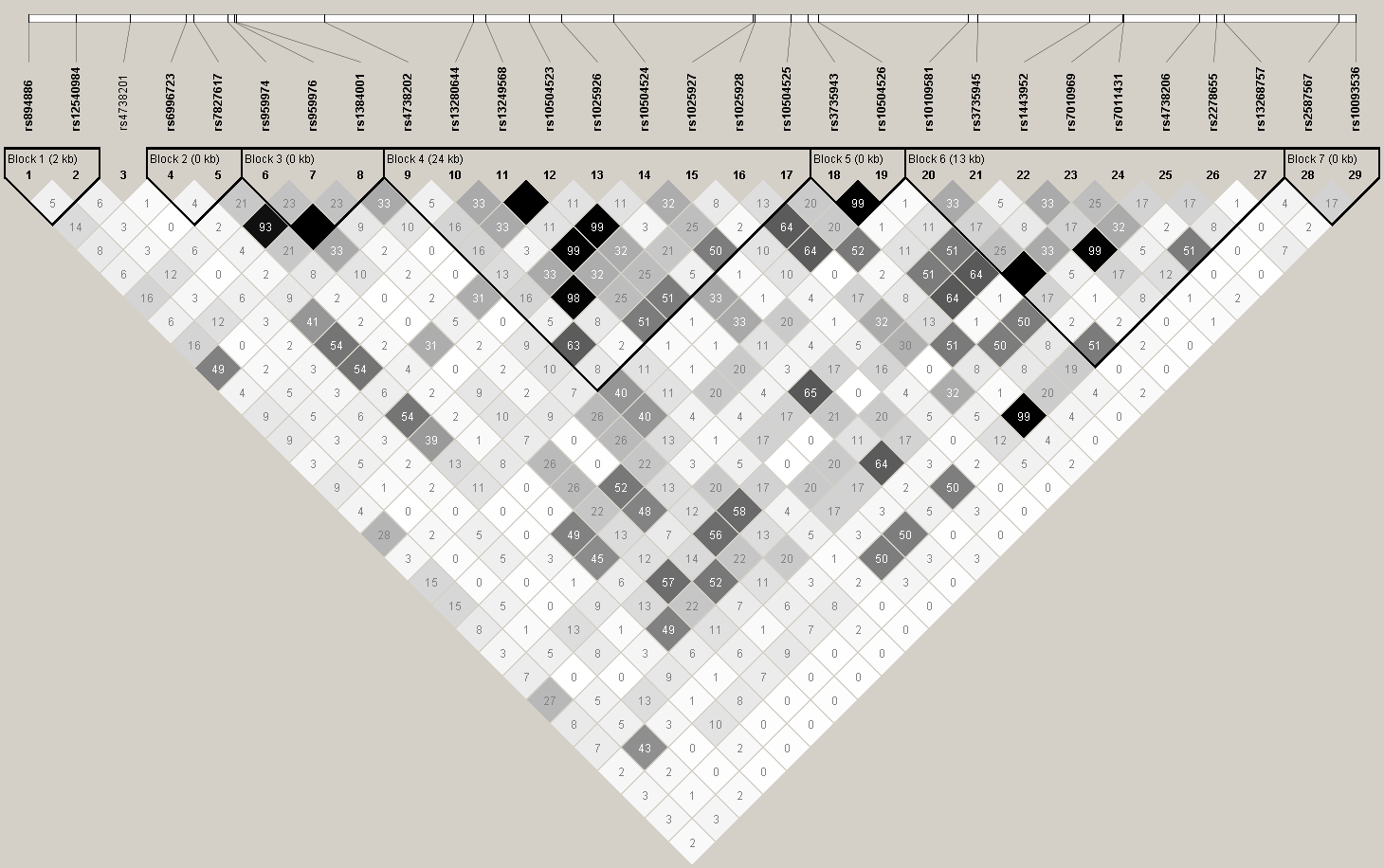


**Figure E5.** Linkage disequilibrium between 29 SNPs in *TRPA1* using the Haploview program [E7]. Values of r^2^ (x100) are shown.


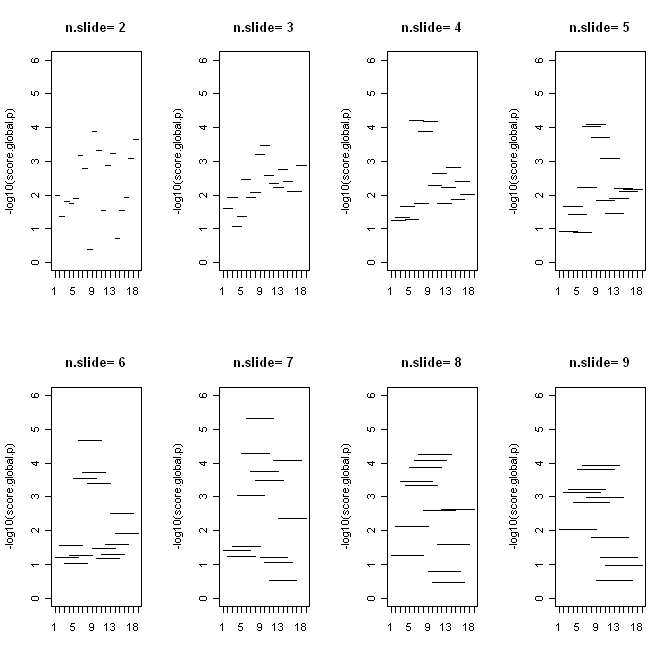


**Figure E6.** Haplotype-based association analysis of *TRPV1* haplotypes and nocturnal cough in 2046 subjects without asthma using a sliding window approach.


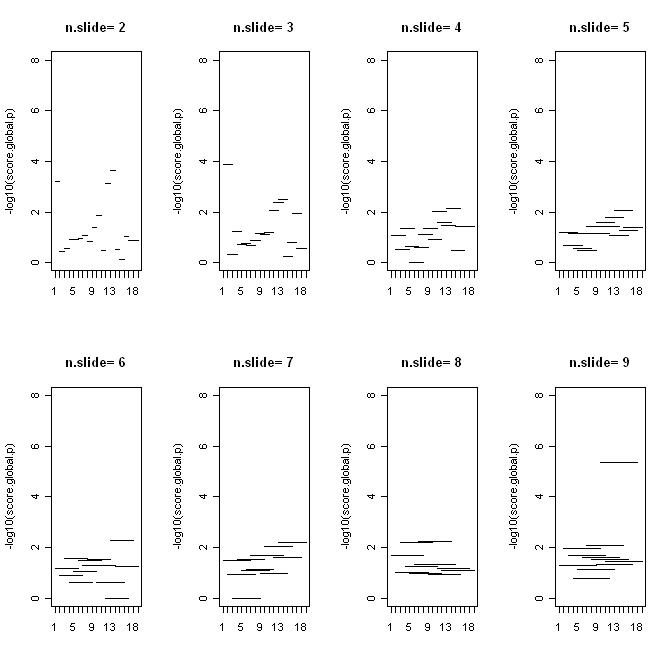


**Figure E7.** Haplotype-based association analysis of *TRPV1* haplotypes and usual cough in 2046 subjects without asthma using a sliding window approach.

**REFERENCES ONLINE SUPPLEMENT**

E1. Kauffmann F, Dizier MH, Pin I, Paty E, Gormand F, Vervloet D, Bousquet J, Neukirch F, Annesi I, Oryszczyn MP, Lathrop M, Demenais F, Lockhart A, Feingold J: **Epidemiological study of the genetics and environment of asthma, bronchial hyperresponsiveness, and atopy: phenotype issues.** *Am J Respir Crit Care Med* 1997, **156**:S123-9.

E2 Moffatt MF, Gut IG, Demenais F, Strachan DP, Bouzigon E, Heath S, von Mutius E, Farrall M, Lathrop M, Cookson WO for the GABRIEL Consortium: **A large-scale, consortium-based genomewide association study of asthma.** *N Engl J Med* 2010, **363**:1211-21.

E3. Benjamini Y, Hochberg Y: **Controlling the False Discovery Rate: A Practical and Powerful Approach to Multiple Testing.** *J R Statist Soc B* 1995, 57:289-300.

E4. Gonzalez JR, Armengol L, Sole X, Guino E, Mercader JM, Estivill X, Moreno V: **SNPassoc: an R package to perform whole genome association studies.** *Bioinformatics* 2007, **23**:644-645.

E5. Lake SL, Lyon H, Tantisira K, Silverman EK, Weiss ST, Laird NM, Schaid DJ: **Estimation and tests of haplotype-environment interaction when linkage phase is ambiguous.** *Hum Hered* 2003, **55**:56-65.

E6. Boos D: **On Generalized Score Tests.** *The American Statistician* 1992, **46:** 327–33.

E7. Barrett JC, Fry B, Maller J, Daly MJ: **Haploview: analysis and visualization of LD and haplotype maps.** *Bioinformatics* 2005, **21**:263-5.
